# EEG power spectral density in locked-in and completely locked-in state patients: a longitudinal study

**DOI:** 10.1007/s11571-020-09639-w

**Published:** 2020-10-23

**Authors:** Arianna Secco, Alessandro Tonin, Aygul Rana, Andres Jaramillo-Gonzalez, Majid Khalili-Ardali, Niels Birbaumer, Ujwal Chaudhary

**Affiliations:** 1 Department of Information Engineering, Bioengineering, Università Degli Studi di Padova, Padua, Italy; 2 Wyss-Center for Bio- and Neuro-Engineering, Chemin de Mines 9, 1202 Geneva, Switzerland; 3 Institute of Medical Psychology and Behavioral Neurobiology, University of Tübingen, Tübingen, Germany

**Keywords:** Resting-state electroencephalogram (EEG), Completely locked-in state (CLIS), LIS (locked-in state), Power spectrum density (PSD), Alpha frequency

## Abstract

Persons with their eye closed and without any means of communication is said to be in a completely locked-in state (CLIS) while when they could still open their eyes actively or passively and have some means of communication are said to be in locked-in state (LIS). Two patients in CLIS without any means of communication, and one patient in the transition from LIS to CLIS with means of communication, who have Amyotrophic Lateral Sclerosis were followed at a regular interval for more than 1 year. During each visit, resting-state EEG was recorded before the brain–computer interface (BCI) based communication sessions. The resting-state EEG of the patients was analyzed to elucidate the evolution of their EEG spectrum over time with the disease’s progression to provide future BCI-research with the relevant information to classify changes in EEG evolution. Comparison of power spectral density (PSD) of these patients revealed a significant difference in the PSD’s of patients in CLIS without any means of communication and the patient in the transition from LIS to CLIS with means of communication. The EEG of patients without any means of communication is devoid of alpha, beta, and higher frequencies than the patient in transition who still had means of communication. The results show that the change in the EEG frequency spectrum may serve as an indicator of the communication ability of such patients.

**Electronic supplementary material:**

The online version of this article (10.1007/s11571-020-09639-w) contains supplementary material, which is available to authorized users.

## Introduction

The cardinal feature of a patient in a locked-in state (LIS) is paralysis of most of the voluntary motor function of the body except the oculomotor function with preserved consciousness (Bauer et al. 1979; Chaudhary et al. 2020a). Because of the preserved oculomotor function and consciousness (Schnakers et al. 2008), patients in LIS have several means of communication (Birbaumer et al. 1999; Wolpaw and McFarland 2004; Kübler et al. 2005; Sellers et al. 2010; Lesenfants et al. 2014; Wolpaw et al. 2018; Tonin et al. 2020). A patient can be in LIS because of the severe brain injury or pontine stroke (Sacco et al. 2008; Sarà et al. 2018; Pistoia et al. 2010; Conson et al. 2010), or progressive neurodegenerative motor neuron disorders (Birbaumer 2006; Birbaumer et al. 2012; Chaudhary et al. 2015, 2016a, b). Amyotrophic lateral sclerosis (ALS) is a severe of all progressive neurodegenerative disorder leading to complete paralysis with symptoms involving both upper and lower motor neurons (Rowland and Shneider 2001). Like any other LIS patient, an ALS patient in LIS are paralyzed with preserved voluntary eye movement control, eye blinks or twitching of other muscles, and intact consciousness. The LIS is not a final state for a patient who has ALS. As the disorder progresses, ALS leads to a state of complete paralysis, including eye movements, transferring patients to the completely locked-in state (CLIS) (Bauer et al. 1979; Chaudhary et al. 2020a). The transition from LIS to CLIS is usually a gradual process that is patient specific. During this transition phase from LIS to CLIS, the patient starts losing their eye movement control and ultimately losing the ability to open their eyes is lost. ianllt, the patients in CLIS have their eyes closed all the time, even in the CLIS, patients are assumed to preserve their cognitive functions (Kübler and Birbaumer 2008).

Many studies have compared electrophysiological signatures from ALS patients and controls (Jayaram et al. 2015; Nasseroleslami et al. 2019; Dukic et al. 2019; Maruyama et al. 2020), reporting features distinguishing the two groups. The most reliable evidence found is a decrease in alpha relative power, with a shift of the peak in the alpha frequency band (generally present in healthy patients’ EEG power spectrum) to lower frequencies (Mai et al. 1998; Hohmann et al. 2018). Several other studies with a different patient population such as depression (Goshvarpour and Goshvarpour 2019), Alzheimer’s disease (Nobukawa et al. 2019), stress (Subhani et al. 2018), autism (Gabard-Durnam et al. 2019), epilepsy (Myers and Kozma 2018) and Parkinson’s disease (Yi et al. 2017) have shown a difference in EEG spectral power, fractal change, power correlation and complexity of resting-state EEG as compared with the healthy participants (Buiza et al. 2018). However, how these features and biomarkers evolve during the ALS progression, reaching a state where they separate patients in different stages of the disease, is still unclear.

This study aims to perform a longitudinal analysis of EEG frequency in three ALS patients, analyzing how the power spectral densities of EEG resting-state recordings evolve in each patient. Two out of three patients considered here are in CLIS (P6 and P9), while the third patient was first in the transition from LIS to CLIS (P11) and, ultimately, in CLIS. The decrease in relative alpha band power is registered in LIS and CLIS patients with respect to controls (Babiloni et al. 2010) (Maruyama et al. 2020), but a direct comparison between these states is still missing. Investigating whether these conditions differ from the electrophysiological point of view can help understand the effects of the transition and possibly monitor the patients for BCI use. In addition, an earlier report on several CLIS patients (Maruyama et al. 2020) needs replication, finding a reduction of higher frequencies in CLIS in a one-session protocol. Whether such a change in spontaneous EEG frequency spectrums indicates functional changes in the central nervous system is now a question of further investigations.

## Materials and methods

The Internal Review Board of the Medical Faculty of the University of Tubingen approved the experiment reported in this study. The study was performed per the guideline established by the Medical Faculty of the University of Tubingen and Helsinki declaration. The patient or the patients’ legal representative gave informed consent. The clinical trial registration number is ClinicalTrials.gov Identifier: NCT02980380.

### Patients

The patients chosen for this study were selected from the available database if the EEG resting-state recordings were in a sufficient number for a longitudinal comparison and covering a time range of at least 1 year. Table 1 lists the most relevant clinical information for each patient and the dates of the acquired EEG recordings.

**Table 1:** List of patients—the table lists for each patient the respective ID, the age and gender, the ALS type diagnosed, a short report of the progression of the disease, and the month and year of visits

Patient ID	Birthday/sex	ALS type	Medical history	Resting state data acquisition date
P6	40/M	Bulbar	2009: Diagnosis Sept 2010: Percutaneous feeding and artificial ventilation Dec 2010: Lost speech and walk 2012: Transition to CLIS	May 2017 September 2017 April 2018 May 2018 January 2019
P9	24/M	Juvenile	2013: Diagnosis Aug 2014: Percutaneous feeding and artificial ventilation 2016: Transition to CLIS	June 2017 November 2017 March 2018 June 2018
P11	35/M	Non-bulbar	Aug 2015: Diagnosis Dec 2015: Lost of speech and walk Jul 2016: Percutaneous feeding and artificial ventilation March 2019: Transition to CLIS	May 2018 August 2018 September 2018 November 2018 December 2018 January 2019 February 2019 March 2019 August 2019 September 2019

### EEG data acquisition

EEG resting-state recordings were acquired during visits to the patients for BCI experiments before the experimental sessions started. From now on, “visits” refers to a period of several subsequent days in which acquisitions were performed. Usually, a single visit lasted for 4 to 5 days, and two subsequent visits were at least 30 days apart from each other.

During the resting state recordings, patients were lying in their beds, being instructed to relax. EEG electrodes were attached according to the 10-5 system, with reference and ground channels placed respectively to their right mastoid and the forehead. EEG signals were recorded using a V-Amp amplifier and active electrodes (Brain Products, Germany). The numbers and positions of electrodes were different between patients and visits due to clinical and experimental needs, as outlined in Supplementary Table 1.

### EEG preprocessing

EEG data were processed using Matlab R2018_b (The MathWorks, Inc., Natick, Massachusetts, U.S.A.) and EEGLAB 14.1.1 (Delorme and Makeig 2004). First, a windowed band-pass filter at 0.5 to 45 Hz was applied to the raw EEG data, followed by down-sampling to 128 Hz. Data were then cleaned from the ocular signal by removing the artifacts using the AAR plug-in (Gómez-Herrero et al. 2006) of EEGLAB. The AAR toolbox process EEG data by first decomposing the time series into spatial components using a Blind Source Separation (BSS) algorithm, then identifying the artifactual components and finally reconstructing the signals using the non-artifactual components. For this study, the decomposition in independent components was obtained through second-order blind identification (SOBI) algorithm (Belouchrani et al. 1997), and artifactual components were automatically identified based on the value of the fractal dimension of the waveform (Gómez-Herrero et al. 2006). In particular, each EEG recording (comprehensive of all the channels acquired) was processed on sliding windows of 180 s, with an overlap period equal to 60 s, and the components with smaller fractal dimensions were selected as artifactual as they correspond to the ones with less low-frequency components. After ocular artifacts rejection was applied singularly to each EEG resting-state record on the complete set of channels, the Cz channel was selected for further analysis.

PSD was obtained through Welch’s overlapped segments averaging estimator, using windows of 5 s length with an overlap of 2 s on a segment of 180 s extracted from the middle of each recording (samples were taken equally before and after the central sample of the complete EEG recording). Then, each PSD was normalized by its median to reduce the effect of different offsets in the recordings. The representative resting-state PSD of each visit was obtained averaging Cz’s PSDs from recordings belonging to the same visit.

**Fig. 1:**
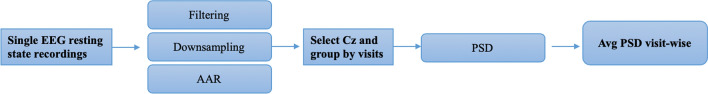
Schematic workflow showing EEG’s processing steps

The relative band-power was then computed from each PSD (for each visit-wise PSD of each patient) to compare relative power values in the three patients quantitatively. The frequency range was divided into delta (0–4 Hz), theta (4–8 Hz), alpha (8–12 Hz), low beta (12–20 Hz), high beta (20–30 Hz), and gamma (30–45 Hz) bands (Fig. 1).

## Results

**Fig. 2:**
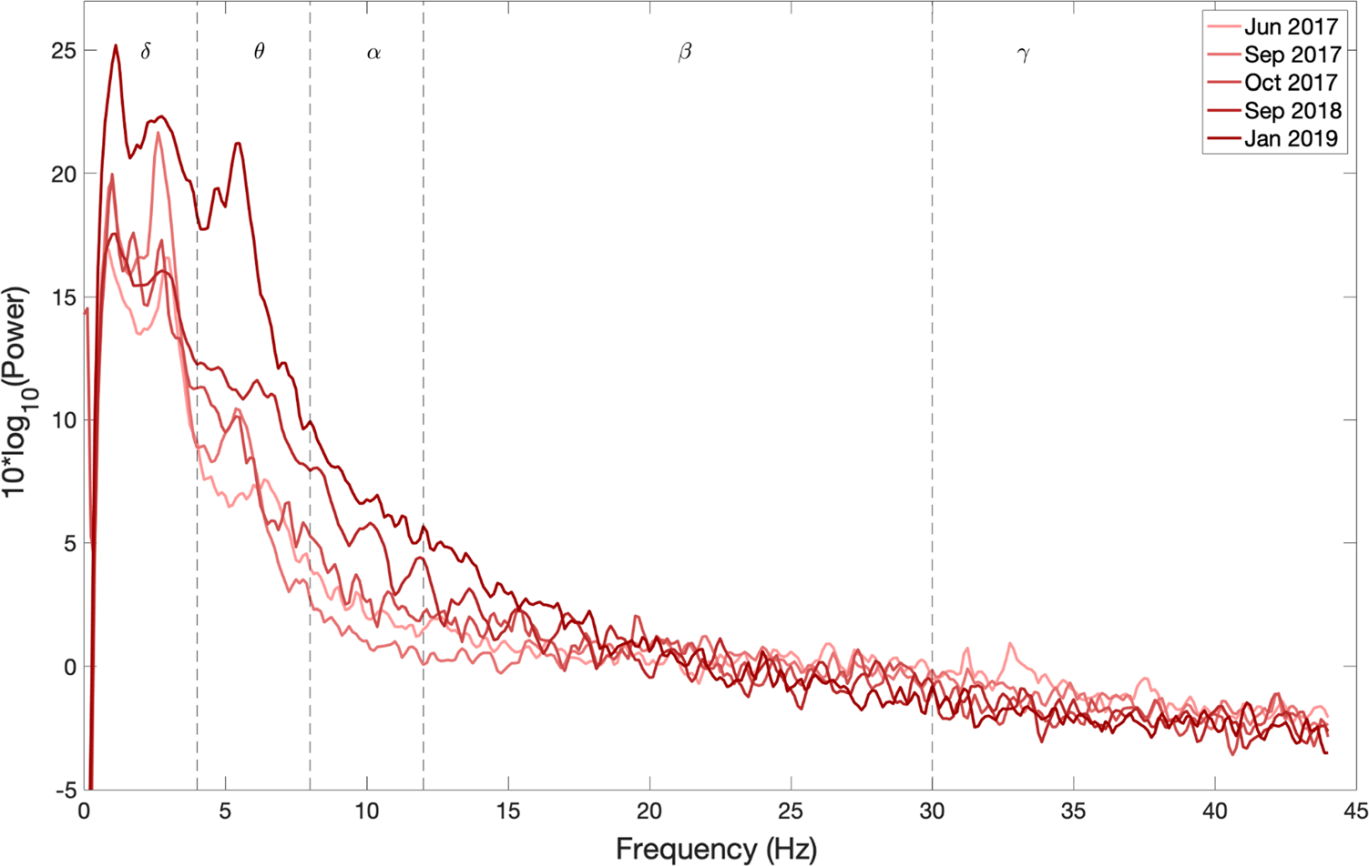
EEG power spectral density evolution in Patient 6. The PSDs corresponding to different visits is shown in different colors, as explained in the box in the top right corner of the figure. The x-axis represents the frequency in Hz. The y-axis represents the normalized amplitude of the power spectral densities on a logarithmic scale. In dashed lines are shown the frequency bands of interest. The frequency range analyzed is divided in the canonical frequency bands, represented in dashed lines in the figures: delta (1 to 4 Hz), theta (4 to 8 Hz), alpha (8 to 12 Hz), beta (12 to 30 Hz) and gamma (30 to 45 Hz)

**Fig. 3:**
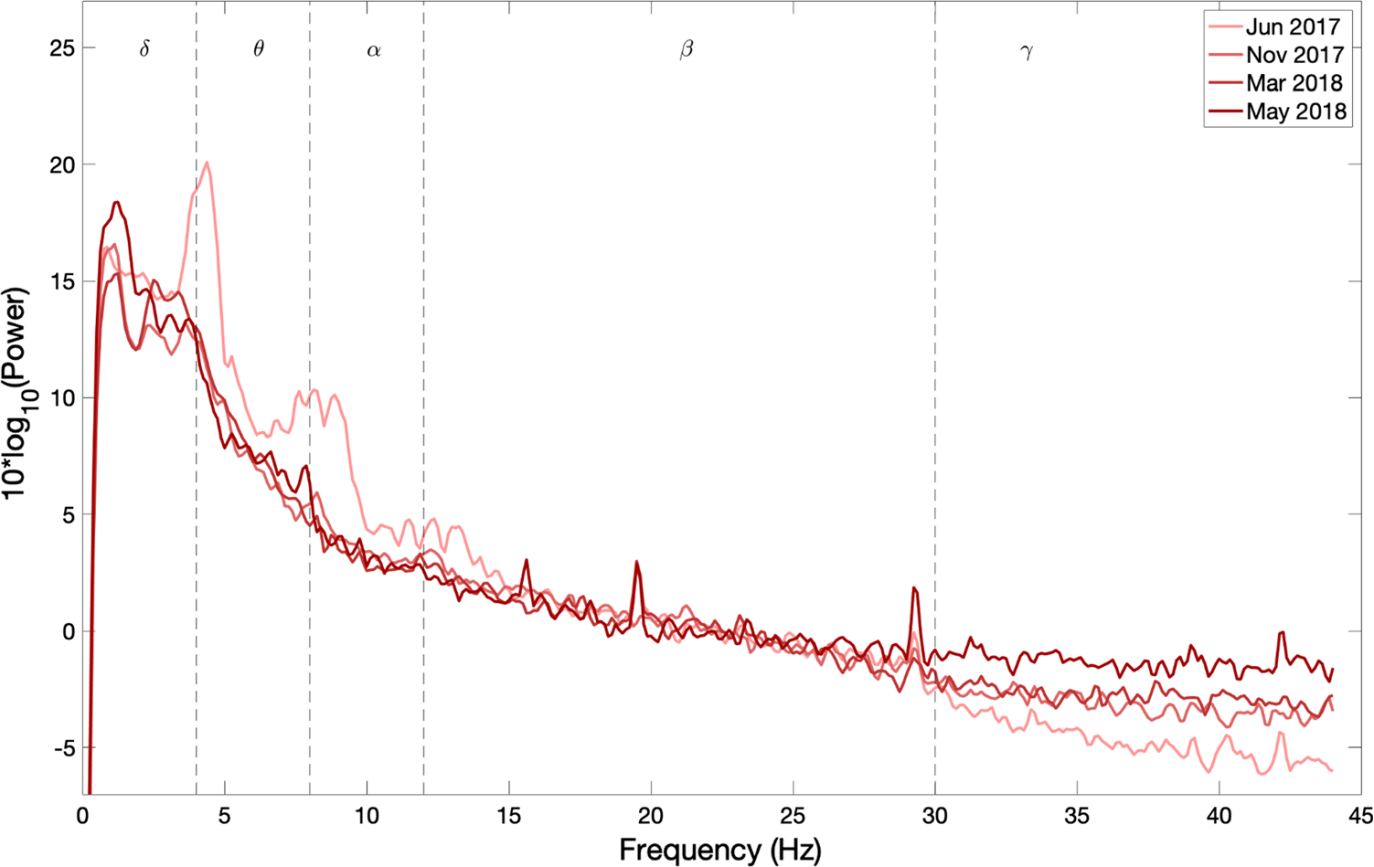
EEG power spectral density evolution in Patient 9. The details of the figure are the same as explained in the legend of Fig. 2

**Fig. 4:**
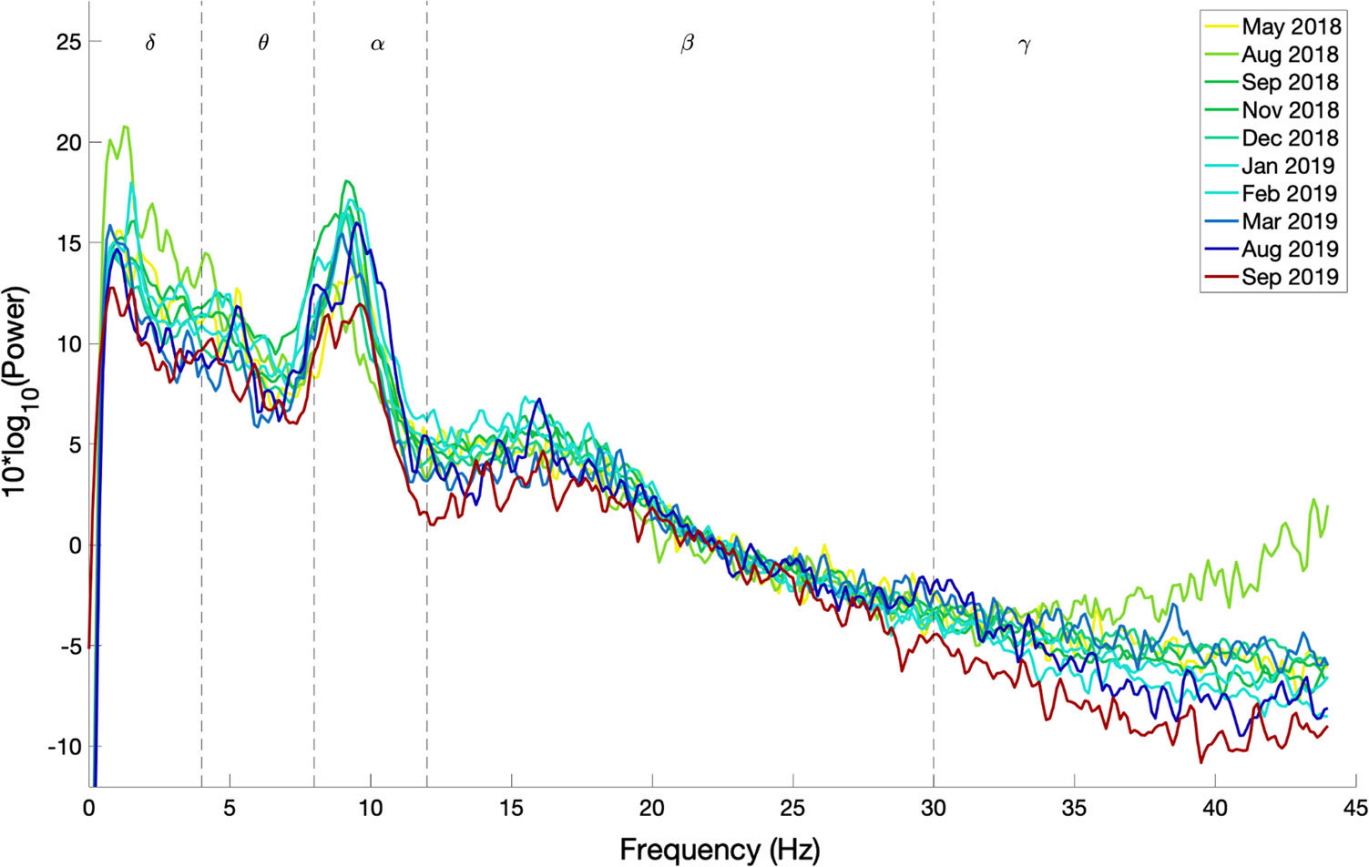
EEG power spectral density evolution in Patient 11. The details of the figure are the same as explained in the legend of Fig. 2

Statistical tests were applied using Matlab 2018b. Pearson’s linear correlation coefficient was computed on subsequent values of relative band-power, obtained for each patient’s set of visits, to investigate the correlation with the corresponding timeline. Then, the Mann–Whitney *U* test was applied to test the power difference between the three patients for each frequency band at the Cz sensor, considering for each of them the whole set of PSDs. The obtained *p* values were corrected through the False Discovery Rate (FDR) using the Benjamini–Hochberg method (Benjamini and Hochberg 1995) to compensate for the multiple comparisons of 6 frequency bands. The results are reported through the visualization of the PSD profile’s evolution within the period of observation for each patient separately. The evolution of PSD of patients 6, 9, and 11 are shown in Figs. 2, 3, and 4, respectively. The results on the variance within visits relative band power and power spectral density of each patient is shown in Supplementary Text 1, where we show that the variance within a visit to be insignificant.

**Fig. 5:**
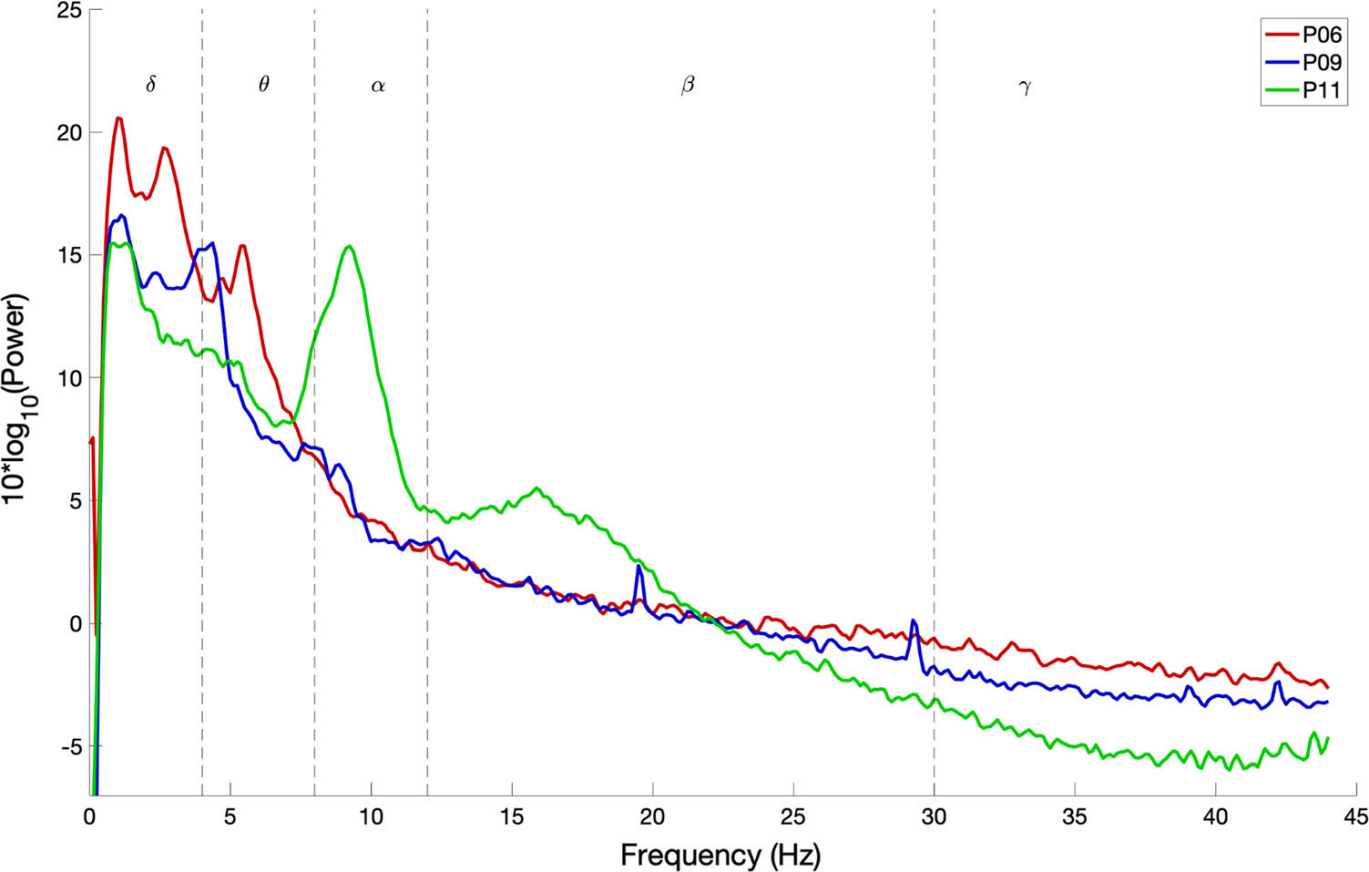
Comparison of average EEG power spectral densities in Patients 6, 9, and 11. The red, blue, and green traces correspond to the average PSDs at electrode Cz for patients 6, 9, and 11, respectively. The x-axis represents the frequency in Hz. The y-axis represents the normalized amplitude of the power spectral densities in the logarithmic scale. In dashed lines are shown the frequency bands of interest as described in the legend of Fig. 2

**Fig. 6:**
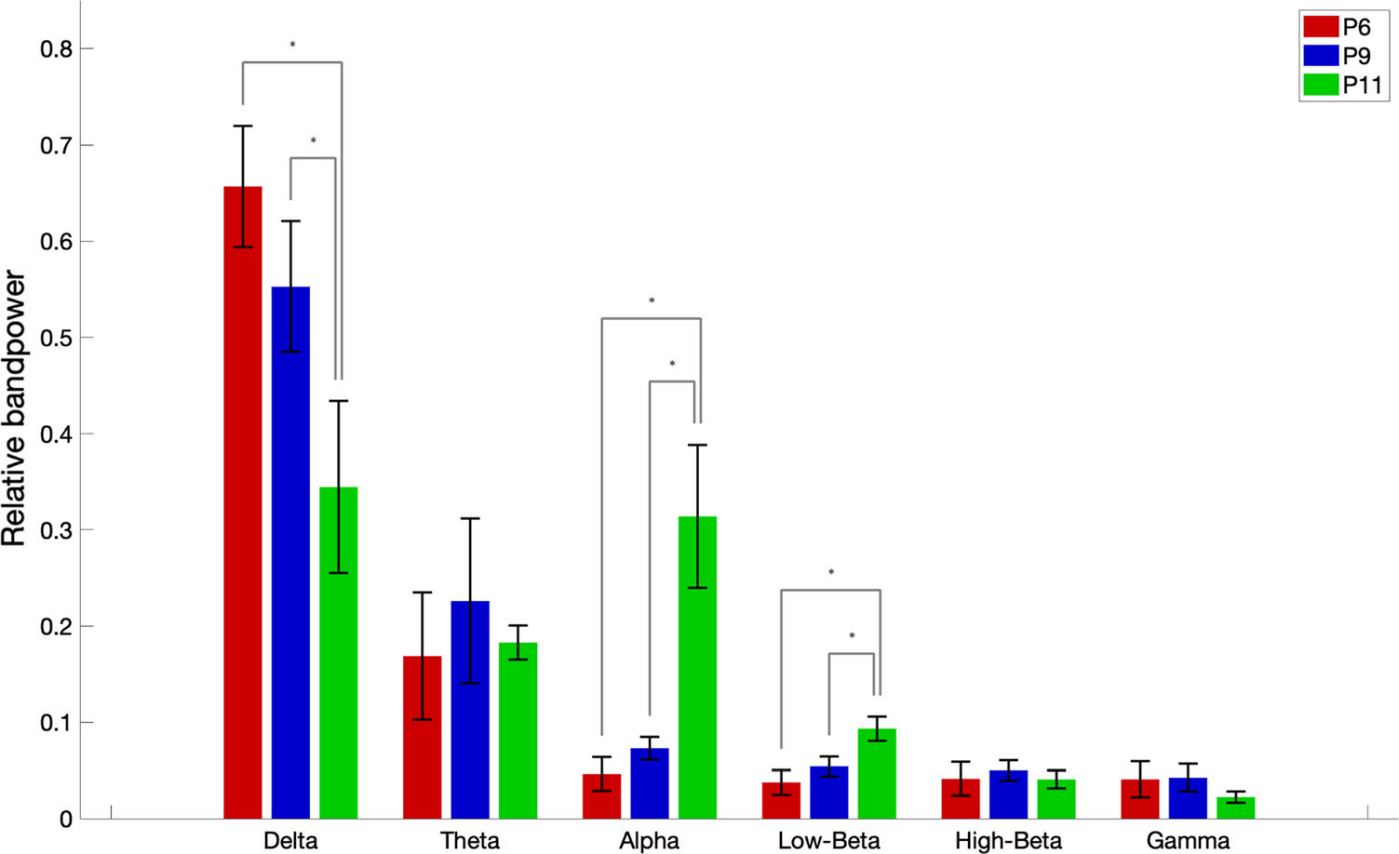
Relative band power at electrode Cz. Error bars represent standard deviations. The figure depicts the significant power differences between patients 6, 9, and 11 in the two-tailed Wilcoxon rank-sum test with False Discovery Rate correction are marked: *p < 0.05. The x-axis represents the different frequency bands in Hz, and the y-axis represents the relative band power

It can be observed from Figs. 2 and 3 that the frequency content of patients 6 and 9, who are in CLIS, are shifted towards delta and theta frequency bands. During the observation period reported in this paper, no general evolution of trends could be seen in patients 6 and 9. While Patient 11 has activity in the alpha band, present in all the recordings within the observation period, as shown in Fig. 4. Nevertheless, a decrease in the power of the EEG signal as the patient transitioned from LIS to CLIS and, ultimately, in CLIS could be observed. The frequency content of patients’ 6 and 9 EEG is very different from the EEG of patient 11. This aspect is more evident in Fig. 5, where the average of the PSDs related to all the visits grouped for patients is presented. These results were confirmed by the results of the Mann–Whitney *U* test shown in Fig. 6, which revealed the significant difference in the relative band power between Patient 11 and the two CLIS patients (Patients 6 and 9) at delta, alpha, and low-beta frequency bands. On the other hand, no significant difference was found over the values of relative power between patients 6 and 9.

## Discussion and conclusion

A longitudinal resting-state analysis of patients in LIS and CLIS reveals a trend on the variation of EEG relative band power within the observation period. Patient 6, who is in CLIS since 2012 and was recorded for the first time in May 2017, shows a stable EEG frequency spectrum with dominant frequency in the delta and theta band. Patient 9, who is in CLIS since 2017 and was recorded for the first in June 2017 also shows a trend similar to patient 6. When we started recording Patient 11 in May 2018, the patient had control over his eye-movements but was unable to communicate with the eye-tracker based communication system because of his inability to fixate his gaze. During every visit to each patient, brain–computer interface (BCI)-based communication was attempted after resting-state recording. With patients 6 and 9, functional near-infrared spectroscopy (fNIRS) based communication was attempted, except for the visit 1 of patient 6, we were not able to establish a reliable means of communication using fNIRS based BCI communication system (Chaudhary et al. 2017) with these two patients. The fNIRS based BCI communication system was employed for patient 6 and 9 because it was demonstrated earlier that EEG-based BCI system had failed so far to provide a means of communication to the patients in CLIS (Kübler and Birbaumer 2008) except for a short one-session period report (Okahara et al. 2018) while fNIRS based BCI communication system showed some promise (Gallegos-Ayala et al. 2014). Since the patient 11 still had eye-movement an electrooculogram (EOG) based BCI communication was developed and implemented to provide a means of communication to the patient. The EOG-based communication by patient 11 is described in Tonin et al. (2020). As described in Tonin et al. (2020), patient 11 was able to employ his eye movement ability to communicate his thoughts and desires until February 2019, albeit with increasing difficulties due to the progressive paralysis of his eye muscles associated with the progression of the amyotrophic lateral sclerosis. From February 2019, the patient 11 could not employ his eye-movement to drive the EOG-based communication system (please refer to (Tonin et al. 2020) for further details). Patient 11 could not communicate reliably with his eyes from March 2019 onwards. He was implanted with microelectrodes in the motor region to provide him a means of communication (Please refer to Chaudhary et al. 2020b for details). The patient although in CLIS was able to form phrases and sentences to express his desires and wishes (Chaudhary et al. 2020b). His EEG spectrum remained constant throughout the observation period reported in this paper.

Patients 6 and 9, although of different ages and being in CLIS for different time periods, have the same EEG spectrum, which is significantly different from patient 11, who was first in LIS, then in the transition from LIS to CLIS and ultimately in CLIS during the period of observation reported in this paper. The main difference between patients 6 and 9 and patient 11 is that since we started following patients 6 and 9, they never had any means of communication. While we were able to provide a means of communication to patient 11 despite his degrading oculomotor function. It can be stated that from the patients reported in this longitudinal analysis, patients without any means of communication have different EEG spectrums than a patient who, despite being in CLIS, has a means of communication. It can also be hypothesized that if a patient has a means of communication despite being in CLIS the general shift in EEG spectrum to the lower bands might not occur, but to generalize these results to other patients in LIS and CLIS with and without means of communication, there is a need to perform such a longitudinal study on the large patient population. Also, a contrary causality is possible: with loss of normal EEG power spectrum and the underlying neurological functionality a loss of communication may be the consequence.

These results are partly supporting an earlier report from our lab of a remarkable reduction of higher frequencies in CLIS (Maruyama et al. 2020), all without any means of communication. It can also be hypothesized that the reason for the failure to establish communication with patients already in CLIS might be due to general shift of their EEG spectrum to the lower bands and absence of alpha and higher frequency bands since all the current EEG based BCI communication systems rely on the alpha and higher frequency bands (Jayaram et al. 2015; Lazarou et al. 2018). Nevertheless, it can also be argued that lack of alpha, in general, might also indicate reduced cognitive processing or compromised vigilance state of the patient (Klimesch 1999). However, in a recent study reported by Khalili-Ardali et al. (2019), patient in CLIS was shown to have the ability to process sentences with motor semantic content and self-related content better than control sentences indicating comprehension and some level of cognitive processing in CLIS in ALS patients. It can also be argued that the patients might be asleep during the period of data acquisition, but recently we showed in a larger sample of patients in CLIS (Malekshahi et al. 2019) that despite a general decrease in their EEG spectrum, patients in CLIS still have an intact sleep and wake cycle.

Thus, there is a need to perform long-term longitudinal studies with patients in LIS, the transition to LIS, and CLIS and parallel cognitive evaluation with BCI assistance to elucidate the evolution in their EEG signature, which afterward may then be used in the development of more efficient non-invasive BCI-systems.

## Electronic supplementary material

Below is the link to the electronic supplementary material.

Supplementary material 1 (DOCX 21 kb)

Supplementary material 2 (DOCX 19 kb)
