# Supplementary material for: EEG power spectral density in locked-in and completely locked-in state patients: a longitudinal study
Source: Cogn Neurodyn. 2020 Oct 23;15(3):473–80. doi: 10.1007/s11571-020-09639-w (PMC8131474; doi:10.1007/s11571-020-09639-w)
Supplement: Supplementary file 1 — Supplementary material 1 (DOCX 21 kb) [file 11571_2020_9639_MOESM1_ESM.docx]

**Supplementary Table 1: Location of electrodes.**

| **Patient** | **Visit** | **Day** | **Set of EEG/EOG channels acquired** |
| --- | --- | --- | --- |
| P6 | Jun 2017 | 1 | 'EOGU, EOGD, EOGL, EOGR, FC5, FC6, C5, C6, Cz, T9, T10' |
|  |  | 2 | 'EOGU, EOGD, EOGL, EOGR, FC5, FC6, C5, C6, Cz, T9, T10' |
|  |  | 3 | 'EOGU, EOGD, EOGL, EOGR, FC5, FC6, C5, C6, Cz, T9, T10' |
|  |  | 4 | 'EOGU, EOGL, EOGR, FC5, FC6, C5, C6, Cz, T9, T10' |
|  |  | 5 | 'EOGUL, EOGDL, EOGUR, EOGDR, FC5, FC6, C5, C6, Cz' |
|  |  | 6 | 'EOGUL, EOGDL, EOGUR, EOGDR, FC5, FC6, C5, C6, Cz' |
|  | Sep 2017 | 1 | 'EOGU, EOGD, EOGR, EOGL, FC2, FC4, FC1, FC3, Cz' |
|  |  | 2 | 'P7, P4, Cz, Pz, P3, P8, O1, O2, T8, C4, F4, C3, F3, Fp1, T7, F7' |
|  |  | 3 | 'P7, P4, Cz, Pz, P3, P8, O1, O2, T8, C4, F4' |
|  |  | 4 | 'P7, P4, Cz, Pz, P3, P8, O1, O2, T8, C4, F4, Fp2, Fz, C3, F3, Fp1, T7, F7' |
|  |  | 5 | 'P7, P4, Cz, Pz, P3, P8, O1, O2, T8, T7' |
|  | Oct 2017 | 1 | 'EOGU, EOGD, EOGR, EOGL, F3, FC3, F4, FC4, Cz' |
|  |  | 2 | 'EOGU, EOGD, EOGR, EOGL, F3, FC3, F4, FC4, Cz' |
|  |  | 3 | 'EOGU, EOGD, EOGR, EOGL, F3, FC3, F4, FC4, Cz' |
|  | Sep 2018 | 1 | 'R1, R2, C2, Cz, C1, Fz, L1, L2, P4, Pz, P3, EOGR, EOGU, EOGL' |
|  |  | 2 | 'R1, R2, C2, Cz, C1, Fz, L1, L2, P4, Pz, P3, EOGR, EOGU, EOGL' |
|  |  | 3 | 'R1, R2, C2, Cz, C1, Fz, L1, L2, P4, Pz, P3, EOGR, EOGU, EOGL' |
|  |  | 4 | 'R1, R2, C2, Cz, C1, Fz, L1, L2, P4, Pz, P3, EOGR, EOGU, EOGL' |
|  | Jan 2019 | 1 | 'AF3, F3, F5, FC3, FC5, C5, C3, T7, CP5, CP3, CP1, C1, Fz, FCz, F4, Cz, FC4,  C4, C2, CP2, CP4, CPz, EMGTLU, EMGTLD, EOGU, EOGD, EMGTRU,  EMGTRD, EMGMDL, EMGMUL, EMGMDR, EMGMUR' |
|  |  | 2 | 'AF3, F3, F5, FC3, FC5, C5, C3, T7, CP5, CP3, CP1, C1, Fz, FCz, F4, Cz, FC4,  C4, C2, CP2, CP4, CPz, EMGTLU, EMGTLD, EOGU, EOGD, EMGTRU,  EMGTRD, EMGMDL, EMGMUL, EMGMDR, EMGMUR' |
|  |  | 3 | 'AF3, F3, F5, FC3, FC5, C5, C3, T7, CP5, CP3, CP1, C1, Fz, FCz, F4, Cz, FC4,  C4, C2, CP2, CP4, CPz, EMGTLU, EMGTLD, EOGU, EOGD, EMGTRU,  EMGTRD, EMGMDL, EMGMUL, EMGMDR, EMGMUR' |
| ‘EOGU’ – EOG electrode placed above eyebrow. ‘EOGD’ – EOG electrode placed below the eye. ‘EOGL’ – EOG placed at the corner of left eye. ‘EOGR’ – EOG electrode placed at the corner of right eye. | | | |
| P9 | Jun 2017 | 1 | 'F3, F4, C3, C4, Cz, EMG, EOGU, EOGD, EOGL, EOGR' |
|  |  | 2 | 'F3, F4, C3, C4, Cz, EMG1, EOGU, EOGD, EOGL, EOGR, EMG2' |
|  |  | 3 | 'F3, F4, C3, C4, Cz, EMG1, EOGU, EOGD, EOGL, EOGR, EMG2' |
|  |  | 4 | 'F3, F4, C3, C4, Cz, EMG1, EOGU, EOGD, EOGL, EOGR, EMG2' |
|  | Nov 2017 | 1 | 'EOGU, EOGD, EOGR, EOGL, F3, FC3, F4, FC4, Cz, EMG, EMGchin' |
|  |  | 2 | 'EOGU, EOGD, EOGR, EOGL, F3, FC3, F4, FC4, Cz, EMG, EMGchin' |
|  |  | 3 | 'EOGU, EOGD, EOGR, EOGL, F3, FC3, F4, FC4, Cz, EMG, EMGchin' |
|  |  | 4 | 'EOGU, EOGD, EOGR, EOGL, F3, FC3, F4, FC4, Cz, EMG, EMGchin' |
|  | Mar 2018 | 1 | 'EOGU, EOGD, EOGR, EOGL, F3, FC3, F4, FC4, Cz, EMG, EMGchin' |
|  |  | 2 | 'EOGU, EOGD, EOGR, EOGL, F3, FC3, F4, FC4, Cz, EMG, EMGchin' |
|  |  | 3 | 'EOGU, EOGD, EOGR, EOGL, F3, FC3, F4, FC4, Cz, EMG, EMGchin' |
|  |  | 4 | 'EOGU, EOGD, EOGR, EOGL, F3, FC3, F4, FC4, Cz, EMG, EMGchin' |
|  |  | 5 | 'EOGU, EOGD, EOGR, EOGL, F3, FC3, F4, FC4, Cz, EMG, EMGchin' |
|  | May 2018 | 1 | 'F4, FC4, F3, FC3, Cz, C1, C2, EOGU, EOGD, EOGL, EOGR, EMGR, EMGL' |
|  |  | 2 | 'F4, FC4, F3, FC3, Cz, C1, C2, EOGR, EOGL, EMG' |
|  |  | 3 | 'F4, FC4, F3, FC3, Cz, C1, C2, EOGR, EOGL, EMG' |
|  |  | 4 | 'F4, FC4, F3, FC3, Cz, C1, C2' |
| ‘EMG1’ – EMG electrode placed on the left side next to the edge of lips. ‘EMG2’ – EMG electrode placed on the right side next to the edge of lips. ‘EOGU’ – EOG electrode placed above eyebrow. ‘EOGD’ – EOG electrode placed below the eye. ‘EOGL’ – EOG placed at the corner of left eye. ‘EOGR’ – EOG electrode placed at the corner of right eye. | | | |
| P11 | May 2018 | 1 | 'EOGU, EOGD, EOGR, EOGL, F4, FC4, F3, FC3, Cz, C1, C2' |
|  | Aug 2018 | 1 | 'AF4, F2, F4, FC2, FC4, Cz, Fz, AF3, F1, F3, FC1, FC3, EOGU, EOGD, EOGR, EOGL' |
|  | Sep 2018 | 1 | 'AF4, F2, F4, FC2, FC4, Cz, Fz, AF3, F1, F3, FC1, FC3, EOGU, EOGD, EOGR, EOGL' |
|  |  | 2 | 'AF4, F2, F4, FC2, FC4, Cz, Fz, AF3, F1, F3, FC1, FC3, EOGU, EOGD, EOGR, EOGL' |
|  |  | 3 | 'AF4, F2, F4, FC4, Cz, Fz, AF3, F1, F3, FC1, FC3, EOGU, EOGD, EOGR, EOGL' |
|  |  | 4 | 'AF4, F2, F4, FC4, Cz, Fz, AF3, F1, F3, FC1, FC3, EOGU, EOGD, EOGR, EOGL' |
|  | Nov 2018 | 1 | 'F2, F4, FC2, FC4, C4, C2, Cz, C1, FC1, FC3, F1, F3, EOGU, EOGR, EOGL' |
|  |  | 2 | 'F2, F4, FC2, FC4, C4, C2, Cz, C1, FC1, FC3, F1, F3, EOGU, EOGR, EOGL' |
|  |  | 3 | 'F2, F4, FC2, FC4, C4, C2, Cz, C1, FC1, FC3, F1, F3, EOGU, EOGR, EOGL' |
|  | Dec 2018 | 1 | 'F2, F4, FC2, FC4, C4, C2, Cz, C1, FC1, FC3, F1, F3, EOGU, EOGD, EOGR, EOGL' |
|  |  | 2 | 'F2, F4, FC2, FC4, C4, C2, Cz, C1, FC1, FC3, F1, F3, EOGU, EOGD, EOGR, EOGL' |
|  |  | 3 | 'F2, F4, FC2, FC4, C4, C2, Cz, C1, FC1, FC3, F1, F3, EOGU, EOGD, EOGR, EOGL' |
|  | Jan 2019 | 1 | 'F2, F4, FC2, FC4, C4, C2, Cz, C1, FC1, F3, F1, Fz, EOGR, EOGL, EOGUR, EOGDR, EOGUL, EOGDL, EOGDiagR, EOGDiagLU' |
|  |  | 2 | 'F2, F4, FC2, FC4, C4, C2, Cz, C1, FC1, F3, F1, Fz, EOGR, EOGL, EOGUR, EOGDR, EOGUL, EOGDL, EOGDiagR, EOGDiagLU' |
|  |  | 3 | 'F2, F4, FC2, FC4, C4, C2, Cz, C1, FC1, F3, F1, Fz, EOGR, EOGL, EOGUR, EOGDR, EOGUL, EOGDL, EOGDiagR, EOGDiagLU' |
|  |  | 4 | 'F2, F4, FC2, FC4, C4, C2, Cz, C1, FC1, F3, F1, Fz, EOGR, EOGL, EOGUR, EOGDR, EOGUL, EOGDL, EOGDiagR, EOGDiagLU' |
|  | Feb 2019 | 1 | 'C1, Cz, C2, F4, FC4, F3, FC3, EOGU, EOGD, EOGR, EOGL' |
|  |  | 2 | 'C1, Cz, C2, F4, FC4, F3, FC3, EOGU, EOGD, EOGR, EOGL' |
|  | Mar 2019 | 1 | 'F4, FC2, FC4, C2, Cz, C1, FC1, FC3, F3, EOGU, EOGD, EOGR, EOGL, EOGDUR, EOGDDL' |
|  | Aug 2019 | 1 | 'EOGU, EOGD, EOGR, EOGL, F3, F4, Cz, C3, C4, PD' |
|  | Sep 2019 | 1 | 'EOGUL, EOGDL, EOGR, EOGL, F3, F4, Cz, C3, C4, PD ' |
| ‘EOGUL’ – EOG electrode placed above eyebrow of left eye. ‘EOGDL’ – EOG electrode placed below the left eye. ‘EOGL’ – EOG placed at the corner of left eye. ‘EOGR’ – EOG electrode placed at the corner of right eye. ‘EOGDiagR’ – EOG electrode placed diagonal to the right eye. ‘EOGDiagLU’ – EOG electrode placed diagonally above the left eye. | | | |
