# Supplementary material for: EEG power spectral density in locked-in and completely locked-in state patients: a longitudinal study
Source: Cogn Neurodyn. 2020 Oct 23;15(3):473–80. doi: 10.1007/s11571-020-09639-w (PMC8131474; doi:10.1007/s11571-020-09639-w)
Supplement: Supplementary file 2 — Supplementary material 2 (DOCX 19 kb) [file 11571_2020_9639_MOESM2_ESM.docx]

**RELATIVE BAND POWER – VARIANCE ANALYSIS**

**PATIENT 6**

|  | **Within visit variance *** | | | | | | |
| --- | --- | --- | --- | --- | --- | --- | --- |
| **Visit** | **Delta** | **Theta** | | **Alpha** | **Low-beta** | **High- beta** | **Gamma** |
| Jun 2017 | 0.0322 | 0.0015 | | 0.0003 | 0.0013 | 0.0072 | 0.0068 |
| Sep 2017 | 0.0174 | 0.0012 | | 0.00022 | 0.00069 | 0.00166 | 0.00117 |
| Oct 2017 | 0.0010 | 0.0034 | | 0.00025 | 4.791e-05 | 2.710e-05 | 6.636e-05 |
| Sep 2018 | 0.0017 | 0.0005 | | 5.374e-05 | 0.0001 | 0.0001 | 0.0005 |
| Jan 2019 | 0.0024 | 0.0079 | | 0.0002 | 0.00018 | 0.0001 | 0.0001 |
|  | **Overall variance **** | | | | | | |
|  | 0.015 | | 0.0044 | 0.0004 | 0.0007 | 0.0029 | 0.0029 |
|  | **Overall variance ***** | | | | | | |
|  | 0.0049 | | 0.0054 | 0.0003 | 0.0002 | 0.0003 | 0.0004 |

**PATIENT 9**

|  | **Within visit variance *** | | | | | | |
| --- | --- | --- | --- | --- | --- | --- | --- |
| **Visit** | **Delta** | **Theta** | | **Alpha** | **Low-beta** | **High- beta** | **Gamma** |
| Jun 2017 | 0.0045 | 0.0125 | | 6.260e-05 | 0.0003 | 0.0003 | 0.0001 |
| Nov 2017 | 0.0025 | 0.0001 | | 0.0002 | 0.0002 | 0.0001 | 0.0001 |
| Mar 2018 | 0.0014 | 0.0002 | | 0.0001 | 0.0002 | 0.0001 | 1.503e-05 |
| May 2018 | 0.0029 | 0.0002 | | 0.0001 | 7.538e-05 | 0.0001 | 0.0002 |
|  | **Overall variance **** | | | | | | |
|  | 0.0065 | | 0.0059 | 0.0004 | 0.0004 | 0.0003 | 0.0003 |
|  | **Overall variance ***** | | | | | | |
|  | 0.0070 | | 0.0077 | 0.0005 | 0.0004 | 0.0004 | 0.0004 |

**PATIENT 11**

|  | **Within visit variance *** | | | | | | |
| --- | --- | --- | --- | --- | --- | --- | --- |
| **Visit** | **Delta** | **Theta** | | **Alpha** | **Low-beta** | **High- beta** | **Gamma** |
| Sep 2018 | 0.003 | 1.8e-4 | | 0.0065 | 6.070e-05 | 7.985e-05 | 1e-4 |
| Nov 2018 | 0.0013 | 8.308e-05 | | 0.0012 | 9.804e-06 | 3.477e-05 | 1.882e-05 |
| Dec 2018 | 0.0007 | 0.0002 | | 0.0041 | 0.0005 | 0.0001 | 0.0001 |
| Jan 2019 | 0.004 | 1.976e-05 | | 0.0075 | 5.195e-05 | 9.298e-06 | 9.841e-06 |
| Feb 2019 | 0.014 | 0.0001 | | 0.0127 | 1.3318e-05 | 2.1145e-05 | 3.1543e-05 |
|  | **Overall variance **** | | | | | | |
|  | 0.0064 | | 0.0004 | 0.0065 | 0.0002 | 0.0001 | 6.9749e-05 |
|  | **Overall variance ***** | | | | | | |
|  | 0.0089 | | 0.0003 | 0.0061 | 0.0002 | 9.647e-05 | 3.753e-05 |

Data from May 2018, Aug 2018, Mar 2019, Aug 2019 and Sep 2019 are excluded from the analysis since they consist of a single EEG recording session only.

* the variance is computed between relative band power values grouped by visit

** the overall variance is computed between relative band power values of all recordings acquired (all days)

*** the overall variance is computed between relative band power values averaged over all visits’ PSD

**POWER SPECTRAL DENSITY – VARIANCE ANALYSIS**

| **Patient** | **Within visit variance** | **Overall variance*** | **Overall variance**** |
| --- | --- | --- | --- |
| **Patient 6** | 1.3327e-05 | 1.404e-05 | 3.984e-06 |
| **Patient 9** | 7.780e-06 | 7.780e-06 | 4.3465e-06 |
| **Patient 11** | 3.960e-06 | 4.515e-06 | 4.1644e-06 |

Within visit variance: average variance of normalized PSD frequency points grouped by visit

Overall variance*: average variance of normalized PSD frequency points of all recordings

Overall variance**: average variance of normalized PSD frequency points from visit’s PSD (average of days’ PSD grouped by visit)

**Patient 6**

| **Jun 2017** | **Sep 2017** | **Oct 2017** | **Sep 2018** | **Jan 2019** |
| --- | --- | --- | --- | --- |
| 1.20126e-05 | 2.305e-05 | 1.849e-05 | 1.249e-06 | 1.182e-05 |

**Patient 9**

| **Jun 2017** | **Nov 2017** | **Mar 2018** | **May 2018** |
| --- | --- | --- | --- |
| 1.055e-05 | 1.965-06 | 3.699e-06 | 6.0903e-06 |

**Patient 11**

| **Sep 2018** | **Nov 2018** | **Dec 2018** | **Jan 2019** | **Feb 2019** |
| --- | --- | --- | --- | --- |
| 2.920e-06 | 2.219e-06 | 2.065e-06 | 3.889e-06 | 8.706e-06 |
